# Mindfulness-based interventions in schools—a systematic review and meta-analysis

**DOI:** 10.3389/fpsyg.2014.00603

**Published:** 2014-06-30

**Authors:** Charlotte Zenner, Solveig Herrnleben-Kurz, Harald Walach

**Affiliations:** Institute for Transcultural Health Studies, European University ViadrinaFrankfurt Oder, Germany

**Keywords:** mindfulness, children, meta-analysis, systematic review, stress, school-age, resilience

## Abstract

Mindfulness programs for schools are popular. We systematically reviewed the evidence regarding the effects of school-based mindfulness interventions on psychological outcomes, using a comprehensive search strategy designed to locate both published and unpublished studies. Systematic searches in 12 databases were performed in August 2012. Further studies were identified via hand search and contact with experts. Two reviewers independently extracted the data, also selecting information about intervention programs (elements, structure etc.), feasibility, and acceptance. Twenty-four studies were identified, of which 13 were published. Nineteen studies used a controlled design. In total, 1348 students were instructed in mindfulness, with 876 serving as controls, ranging from grade 1 to 12. Overall effect sizes were Hedge's *g* = 0.40 between groups and *g* = 0.41 within groups (*p* < 0.0001). Between group effect sizes for domains were: cognitive performance *g* = 0.80, stress *g* = 0.39, resilience *g* = 0.36, (all *p* < 0.05), emotional problems *g* = 0.19 third person ratings *g* = 0.25 (both n.s.). All in all, mindfulness-based interventions in children and youths hold promise, particularly in relation to improving cognitive performance and resilience to stress. However, the diversity of study samples, variety in implementation and exercises, and wide range of instruments used require a careful and differentiated examination of data. There is great heterogeneity, many studies are underpowered, and measuring effects of Mindfulness in this setting is challenging. The field is nascent and recommendations will be provided as to how interventions and research of these interventions may proceed.

## Introduction and background

The application of Mindfulness-Based Interventions (MBIs) has become increasingly popular in the last few years, both in research and practice. Mindfulness can be defined as the psychological capacity to stay willfully present with one's experiences, with a non-judgemental or accepting attitude, engendering a warm and friendly openness and curiosity (Kabat-Zinn, 2005).

Originally derived from eastern traditions and Buddhist psychology, mindfulness can be cultivated by various techniques (Bankart, 2003; Wallace and Shapiro, 2006). Formally, it is trained by meditation practices such as sitting meditation, or physical movement such as yoga or tai chi. These techniques help steady the mind and train its attentional capacity, while also increasing its breadth of focus. Practitioners are instructed to focus their attention on the present moment using an “anchor,” for instance, the breath. When the mind drifts away, the focus is gently brought back to the present moment experience. The practitioner tries to simply observe his or her experience of the present moment without judging or modifying it.

Roughly 30 years ago, Jon Kabat-Zinn introduced mindfulness as a resource into clinical research and practice through the Mindfulness-Based Stress Reduction Program (MBSR). The MBSR program consists of 8 weekly sessions of 2½ h, and a day of mindfulness. Mindfulness is practiced formally in sitting meditation, by simple yoga movements, and in the body-scan, which is a gradual sweeping of attention through the body. Mindfulness is also cultivated in daily activities such as eating, and by using it as a resource in emotionally challenging situations or in dealing with physical pain. The recommended daily home practice lasts approximately 45 min, and includes formal and informal exercises. Moreover, the program includes psycho-education, and attitudes such as not judging, a beginner's mind, trust, non-striving, acceptance, letting go, and patience are encompassed (Kabat-Zinn, 1982, 1990, 2003). The MBSR program became the parent to several variations, such as Mindfulness-Based Cognitive Therapy (MBCT; Segal et al., 2002), initially developed for preventing relapse of depression. In other cognitive-behavioral therapies, such as acceptance and commitment therapy, (ACT; Hayes et al., 1999) and dialectical behavior therapy (DBT; Linehan, 1993), the emphasis of treatment lies on acceptance as well as on change.

In several reviews and meta-analyses, MBIs proved to be effective in a wide range of stress related and clinical problems and disorders for various disease groups (Grossman et al., 2004; Fjorback et al., 2011; Piet and Hougaard, 2011; Piet et al., 2012). In addition, an interesting aspect of MBIs is their potential preventive and health promoting capacity in non-clinical populations: reducing stress, increasing well-being and strengthening immune functions (Davidson et al., 2003; Chiesa and Serretti, 2009; Eberth and Sedlmeier, 2012); promoting personal development such as self-compassion, empathy and perspective taking (Shapiro et al., 1998, 2007; Birnie et al., 2010); increasing attentional capacity (Jha et al., 2007; Tang et al., 2007) and the temporal window of attention (Sauer et al., 2012).

One potential mechanism could be through decreasing the tendency to avoid unwanted experiences, thus generally improving positive affect (Sauer et al., 2011a,b). Mindfulness seems to be the opposite of mind-wandering (Smallwood and Schooler, 2006). Mind-wandering has been linked to the activity of the default-mode network (DMN), i.e., those areas of the brain that become active when the cognitive system remains idle (Raichle et al., 2001). Interestingly, experienced Zen meditators show reduced baseline activity of the DMN (Pagnoni et al., 2008). Since a higher activity of the DMN is related to increased negative affect and to the rate of mistakes in attentional and other tasks (Smallwood et al., 2011), it seems natural that reducing mind-wandering and improving attentional capacities could be beneficial in many respects, and might be one of the generic mechanisms through which mindfulness-based approaches work (Carmody, 2009).

Given the diverse usefulness and beneficial record of MBIs for adults, researchers and clinicians are striving to develop adaptations for children and youths. Research is in its infancy, but initial reviews suggest that MBIs are feasible with children and adolescents and seem to be beneficial in both clinical and non-clinical samples (Black et al., 2009; Burke, 2009). They have been successfully applied to adolescents with attention deficit hyperactivity disorder (ADHD) symptoms (Van der Oord et al., 2012; Weijer-Bergsma et al., 2012), and to adolescents with a variety of externalizing disorders (Bögels et al., 2008). MBIs lead to a reduction in symptoms of depression in minority children (Liehr and Diaz, 2010) and to a reduction in anxiety and increase of social skills in students with learning disorders (Beauchemin et al., 2008). In a study of “at-risk” and HIV-positive youth, decreases in hostility and general and emotional discomfort have been reported, while qualitative data indicated improvements in academic performance, interpersonal relations, stress-reduction, and physical health (Sibinga et al., 2011). Also, first conceptual frameworks have been created as to why MBI's are beneficial for children and youth and how mechanisms might work (Mind and Life Education Research Network (MLERN), 2012; Zelazo and Lyons, 2012).

School appears to be an appropriate setting for such interventions, since children spend a lot of time there and interventions can be brought directly to groups of children in areas of need as part of a preventive approach at little cost (Weare and Nind, 2011). Mindfulness can be understood as the foundation and basic pre-condition for education. Children need to learn to stop their mind wandering and regulate attention and emotions, to deal with feelings of frustration, and to self-motivate. Mindfulness practice enhances the very qualities and goals of education in the 21st century. These qualities include not only attentional and emotional self-regulation, but also prosocial dispositions such as empathy and compassion, self-representations, ethical sensitivity, creativity, and problem solving skills. They enable children to deal with future challenges of the rapidly changing world, ideally becoming smart, caring, and committed citizens (Shapiro et al., 2008; Mind and Life Education Research Network (MLERN), 2012).

Concurrently, reports of increasing clinical problems in children, stress-related problems and problems related to social pressure in and outside school are worrying. Children and youth frequently experience stress in school (Currie et al., 2002; Lohaus and Ball, 2006; Card and Hodges, 2008), which has an impact on the brain structures involved in cognition and mental-health (Lupien et al., 2009). Serious mental disorders are also widespread among children. It has been reported that 21% of the 13 to 18 year olds in the US are currently suffering, or have at some point during their life suffered, from a severe mental disorder (Merikangas et al., 2010), with ADHD, behavioral or conduct problems, anxiety, and depression being the most prevalent current diagnoses (US Department of Health and Human Services, and Centers for Disease Control and Prevention, 2013).

Formal education should always consider the mental health and balance of children. A growing body of research shows that “academic achievement, social and emotional competence and physical and mental health are fundamentally and multiply interrelated. The best and most efficient way to foster any of those is to foster all of them” (Diamond, 2010, p. 789). Schools are therefore confronted with the task of not only being institutions for formal education, but also a place that provides tools for preventing disorders and fostering personal development and well-being in children. These needs have driven educators, teachers, and psychologists to seek methods to improve school-based learning and the social experience connected with it. MBIs in schools are seen as an approach to tackle these challenges, because prevention and education can be provided simultaneously, addressing a wide range of needs and unfulfilled potentials of students.

As a result, various mindfulness programs for schools have been developed and applied within the past few years (see Meiklejohn et al., 2012 for an overview). Several research institutes and associations, such as the Garrison Institute, are initiating workshops and conferences on Mindfulness in Education on a regular basis. Within mailing lists administrated by the *Mindfulness in Education Network* (www.mindfuled.org) or the *Association of Mindfulness in Education* (www.mindfuleducation.org), clinicians, educators, and researchers from all over the world share ideas, material and experiences of mindfulness in schools. The increasing amount of meetings, books, and newspaper articles indicate that the integration of mindfulness into education is received with great interest and is seen as a potentially plausible, cost-effective, and promising approach.

The number of studies evaluating MBI's in school settings is also growing. However, others point out that, to date, enthusiasm about the integration of MBI's in schools surpasses evidence (Greenberg and Harris, 2011). The diversity of programmes and outcome measures combined with the pilot-character of most studies make it difficult to get a general impression of effectiveness, and directions of further research cannot be easily derived. Presenting a narrative review on the literature, Meiklejohn et al. (2012) made a good start summarizing the research published to date, but a quantitative synthesis exclusively integrating studies on MBI's in school context is still lacking. Specifically, it would be helpful to know if there are specific domains in which MBI's are particularly beneficial. At this point the inclusion of unpublished literature, such as doctoral theses, would enrich the discussion, as these often contain supplementary information that could be valuable and could introduce new approaches to this specific research field, such as, for example, the choice of measures. Also, little is known about the feasibility of integrating MBI's into school-routine, for example, the acceptability of different programme elements.

To help progress this field of research, we decided to carry out a meta-analytic review. Aiming to give a complete insight into the actual state of the art, we adopted a very open and comprehensive stance by locating as many studies as possible, both published and unpublished, and by including all relevant material. First, we addressed the types of mindfulness interventions that have been applied and the measures used in order to provide a transparent overview of the field. Second, we explored how MBI's work in a school setting: collecting findings on feasibility and acceptability. With a view to provide recommendations for future research, third, we ascertained the quality of the existing trials and identified possible methodological challenges. Fourth, we carried out a quantitative synthesis in order to ascertain whether effect sizes warrant pursuing this line of research further. By also deriving domain-specific effect sizes, we aimed to clarify the diversity of outcome measures and to address the issue of which domains might be most beneficial for school children.

Since the work was exploratory, it was intended to give orientation and develop further hypotheses rather than to test them. In the following, we present a systematic review of the literature and a meta-analysis of the available information.

## Methods

### Search strategy

A comprehensive search strategy was chosen in order to locate both published and unpublished studies. In August 2012 systematic searches were performed in 12 databases and catalogs including Web of Knowledge, SciVerse Hub, PsychARTICLES, PSYNDEX, Psychology and Behavioral Sciences Collection, ERIC, FIS, The DART-Europe E-Theses Portal, PDQT Open, DissOnline, Openthesis, and UMI Dissertation Express. Mindfulness_ was used as the key word, combined with School_, Classroom_, or Education_, where appropriate. Studies were searched from the first year the database was available and no language restrictions were applied.

After removal of duplicates and screening abstracts of the remaining studies, full-text articles of relevant studies were retrieved for examination. The reference lists of the selected articles were inspected and authors of relevant studies were contacted. Emails were sent to the mailing list of *Mindfulness in Education Network* and the *Association of Mindfulness in Education* in October 2012. All volumes of the *Mindfulness Research Monthly* Newsletter and *Mindfulness* Journal were screened up to and including October 2012.

The first two authors independently extracted the data from the original reports in order to decide on inclusion. Disagreements were solved by discussion.

### Inclusion criteria

Studies were selected if the following criteria were met:

1. Interventions were mindfulness-based.
2. Implementation took place in a school-setting.
3. Participants were pupils or students from grade 1 to 12.
4. Outcomes were quantitative data, referring to psychological aspects.

We sought interventions based on the concept of mindfulness, with classical mindfulness practices such as mindful breathing or the body scan as core elements. Combinations with other methods, such as massage, imaginary journey, or games, were accepted as long as their implementation was aimed at cultivating mindfulness, making it easily accessible for the target age-group and setting. Approaches combining mindfulness and other established techniques such as Autogenic Training or Progressive Muscle Relaxation were excluded, because outcomes cannot clearly be attributed to mindfulness. For the same reason evaluations of trainings mainly based on concentrative meditation, such as Transcendental Meditation, were also excluded. No further methodological exclusion criteria were applied.

### Data extraction

Data on methodology and outcomes of included studies were extracted and coded by the first author and checked by the second author. These data covered information on schools and participants, sample size and study design, applied measures, type of statistical analysis and major findings reported, as well as data necessary for calculating effect sizes. Relevant information concerning interventions and feasibility was extracted by the second author and checked by the first author. This information included setting, structure, and elements of intervention and various aspects of feasibility (e.g., acceptability, fidelity, attrition). In cases where important information was missing, study authors were contacted.

### Statistical methods

The weighted mean effect size (ES) *g* was chosen as a statistic for final analysis. Hedges's *g* is a variation of Cohen's *d* (Cohen, 1988), standardizing the mean difference by a pooled standard deviation using *n*-1 for each sample (Hedges and Olkin, 1985).

(1) $$g_{hedges}\text{​​}=\text{​}\frac{M_{1}-M_{2}}{s_{pooled}}\text{ with }s_{pooled}\text{​}=\text{​}\sqrt{\frac{\left(n_{1}-1\right)s_{1}^{2}+\left(n_{2}-1\right)s_{2}^{2}}{n_{1}+n_{2}-2}}$$

ESs were then multiplied with c(*m*), a correction factor to correct potential bias due to small sample sizes.

(2) $$c(m)=1-\frac{3}{4m-1}$$

where *m* refers to degrees of freedom used to estimated *s*_*pooled*_. (Hedges, 1981). Hedges's *g* can be interpreted according to Cohen's ES conventions (1988) as small (0.2), medium (0.5), and large (0.8).

Within-group ES were calculated for all relevant measures in every study. For controlled trials ES of baseline equivalence and differences in change scores were also derived.

In several cases means and standard deviations were not reported. If statistics like partial eta-squared (interpreted as *r*^2^), *t*- or *F*-values were given, *g* could be derived according to specific formulas. In other cases, all essential data were missing and authors did not provide them after being contacted. In order to prevent bias due to missing data, ES were estimated in alternative ways (marked with a #). Lacking means, for example, could be derived from graphs (8, 14). Missing *SD*s for within-group differences were estimated by deriving standard error of change score differences (8), or were derived from *SD* of within-group differences, assuming that population variance at time 1 and 2 was equal (18). In another study, standard deviations of the norm sample were used for ES calculation (22). If no information was neither reported nor could be extracted, results were suggested to be insignificant and thus ES were estimated as 0 (Rosenthal, 1995). This was done for study no. 8, 12, 18, and 22 (see Table 1).

**Table 1:** **Empirical studies on MBI's in a school-setting**.

**Study**	***N***	**Age range, mean (*SD*), grade and gender**	**School/participant description (country)**	**Study design**	**Measures and domain**		***g*_Hedges_ Baseline equivalence**	***g*_Hedges_ Within-group**	***g*_Hedge_ Differences in change scores**	**Reported findings according to authors**
**RANDOMIZED CONTROLLED TRIALS**										
1. Desmond and Hanich, 2010	40	11–12, 6th grade	Urban, public middle school, low income (USA)	M-group (*n* = 15) vs. C (*n* = 25)	BRIEF (teacher)	**T**	0.26	0.04	0.31	MANOVAs: No sig. time by group interaction (all *p*s > 0.05). Multiple regression analysis: Sig. interaction between pre-test score and group membership for predicting differences in one of eight subscales, indicating that M-group showed greater improvement in ability to shift (*p* < 0.05). In general, M-group maintained or improved executive function skills, while C shows a decline.
		41% female								
2. Flook et al., 2010	64	7–9	On-campus university elementary school, diverse ethical backgrounds (USA)	M-group (*n* = 32) vs. C (*n* = 32)	BRIEF (teacher)	**T**	0.31	0.20	0.08	MANCOVAs with post-test scores as outcome variables: No sig. group main effect, indicating no group differences for pre- to post-test (*p* < 0.05). Sig. interaction between baseline levels and group in teacher report (*p* = 0.005) as well as in parent report (*p* = 0.020). In M-group, children with poorer initial executive function showed greater improvement at Time 2 compared to C.
		8.23 (0.66) 2nd + 3rd grade			BRIEF (parent)	**T**	0.27	0.39	0.12	
		55% female								
3. Franco Justo, 2009	60	15–18	3 public secondary schools (Spain)	M-group (*n* = 30) vs. waitlist c (*n* = 30), follow-up after 3 months	TTCT (verbal)	**C**				Independent and dependent *t*-Tests: Sig. improvement from pre- to post-test in M-group in all subscales (Fluency, Flexibility, Originality; all *p*s < 0.01) and no improvement in C (all *p*s > 0.05). At post-test M-group shows significantly higher scores in all subscales compared to C (all *p*s < 0.01). Effects sustained at follow up compared to pre-test (all *p*s = 0.001), but not compared to post-test (all *p*s > 0.05).
		17.3			-Fluency		−0.11	1.50	1.48	
		1st + 2nd year high school			-Flexibility		0.05	1.53	1.87	
		72% female			-Originality		−0.05	1.61	1.67	
4. Franco Justo et al., 2011a	61	16–18	3 compulsory secondary schools, public (Spain)	M-group (*n* = 31) vs. waitlist c (*n* = 30) Schools were allocated at random	Grades	**C**	−0.27	1.52	1.43	Dependent and independent *t*-Tests: Sig. improvement from pre- to post-test in M-group in all measures (all *p*s = 0.001) and no improvement in C (all *p*s > 0.05). Sig. difference between groups in post-tests (all *p*s > 0.01). Detailed analysis: students with middle range academic performance show the most improvement in Grades (Cohen's *d* = 3.05), Students with low self-concept show most improvement in self-concept (*d* = 5.12), students with high state anxiety benefited the most on state anxiety (*d* = 1.95) and students with medium trait anxiety benefited the most on trait anxiety (*d* = 1.44).
		16.75 (0.83)			Self-concept	**R**	0.59	1.55	1.84	
		1st year high school			STAI	**E**	0.35	0.62	0.11	
		48% female								
5. Franco Justo et al., 2011b	84	16–19	Various compulsory secondary schools (Spain)	M-group (*n* = 42) vs. waitlist C (*n* = 42)	AURE	**R**	−0.06	1.26	1.29	Dependent and independent *t*-Tests: Sig. improvement from pre- to post-test in M-group for all 3 subfactors (*1. Approaching and Coping with a Task 2. Self-Concept and Self-Esteem 3. Empathy and Social Relations;* all *p*s < 0.05) and no improvement in C (all *p*s > 0.05). Sig. difference between groups in post-tests in the first 2 subfactors (*p*s < 0.001), but not in the third (*p* = 0.16).
		17.06 (2.44)								
		1st + 2nd year high school								
		72% female								
6. Mai, 2010	12	13–17	Urban high school, low socio economic status, low performing (USA)	M-group (*n* = 7) vs. waitlist C (*n* = 5), follow-up after 6 weeks	DERS	**E**	0.57	−0.06	−0.60	ANOVAs (repeated measures): No sig. findings were found (all *p*s > 0.05).
		14.4			BRIC (teacher)	**T**	−0.12	−0.10	−0.10	
		(*Mdn* = 14.0), 9th grade, 25% female			Grades	**C**	−0.55	0.02	0.30	
					School attendance	–	−0.05	0.29	0.10	
7. Mendelson et al., 2010	97	10.15 (0.7), 4th + 5th grade	4 urban public elementary schools, low income neighborhood with high levels of violence (USA)	2 M-groups (*n* = 42–47) vs. 2 waitlist C (*n* = 40–43)	PANAS	**R**	−0,14	0.17	0.23	Multiple regressions: M-group demonstrated sig. improvements on the overall scale of Involuntary Engagement compared to C (*p* < 0.001). Sig. differences were found on three of the five subcales (Rumination, Emotional Arousal, Intrusive Thoughts: *p* < 0.05) and a trend for Impulsive Action and Physiologic Arousal (boths *p*s < 0.07). No other sig. results were found. However, depressive symptoms and negative effect displayed a pattern consistent with predictions.
		61% female		4 schools were allocated at random	SMFQ—C	**E**	0.9	0.14	0.02	
					PIML	**R**	−0.21	−0.02	0.09	
					Involuntary Engagement (RSQ)	**S**	0	0.41	0.90	
8. Napoli et al., 2005	194	1st-3rd grade	2 elementary schools (USA)	M-group (*n* = 97) vs. C (*n* = 97)	ACTeRS (teacher)	**T**	^#^	0.20^#^	0.24	*T*-Tests for change scores between groups: Sig. improvement for M-group on attention and social skills subcale of ACTeRS (both *p*s = 0.001). Sig. reduction of Test Anxiety in M-group (*p* = 0.007). Sig. improvement of M-group on selective attention (*p* < 0.001) but not on sustained attention subscale (*p* = 0.350).
					TAS	**E**	^#^	0.38^#^	0.39	
					Selective Attention (TEA-Ch)	**C**	^#^	0.48^#^	0.60	
					Sustained Attention (TEA-Ch)	**C**	^#^	0^#^	0.13	
9. Potek, 2012	30	14–17	2 high schools in an urban or rural setting, diverse range of socioeconomic status (USA)	M-group (*n* = 16) vs. waitlist C (*n* = 14)	MASC	**E**	0.01	1.12	0.85	Repeated-measures ANOVAs: Sig. interaction between time and group on MASC scores (*p* < 0.0001), indicating that the anxiety level of M-group decreased more compared to C. No sig interaction effect on DERS and PSS scores (boths *p*s = 0.14).
		15 (0.98)			DERS	**E**	0.32	0.27	0.33	
		9th-12th grade			PSS	**S**	0.25	0.49	0.42	
		48% female								
10. White, 2012	155	8–11	Public schools, 85% reported having no family stress or health problems, majority of parents went to college (USA)	M-group (*n* = 70) vs. waitlist C (*n* = 85)	FBS	**S**	0.16	−0.17	−0.11	Repeated-measures ANOVAs: Sig. time by group interaction on the SCSI subscale frequency of coping (*p* < 0.04), suggesting that M-group is coping more frequently after intervention. No sig. interaction for Global self-worth (*p* = 0.57) and an approached significance for FBS (*p* = 0.06), indicating increasing stress levels in M-group after intervention compared to C. Further analysis revealed that this was due to a sig. interaction for the stress appraisal subscale of FBS (*p* = 0.005). Compared to C, M-group was more likely to increase their appraisal of stress at post-test.
		9.9 (0.72)			SCSI	**S**	−0.05	0.05	0.16	
		4th + 5th grade			Global Self-worth Scale (SPPC)	**R**	0	0.17	−0.18	
		100% female								
**QUASI-RANDOMIZED CONTROLLED TRIALS**										
11. Broderick and Metz, 2009	122	16–19	Suburban, private catholic high school for female (USA)	M-group (seniors, *n* = 105, age: *M* = 17.43) vs. C (juniors, *n* = 17, age: *M* = 16.41)	PANAS	**R**	−0.21	0.24	0.55	*T*-Tests for change scores between groups: M-group demonstrated sig. reduction in neg. affect and sig increase on the calm/relaxed/self-accepting scale (both *p*s < 0.05). No other measures showed sig. differences in gain scores (*p* > 0.05).
		M-group: Seniors 17.43 (0.53)			Calm/relaxed/self-accepting scale	**R**	0.03	0.33	0.55	Dependet *t*-tests: M-group showed sig. decline in neg. emotions and somatic complaints, sig. increase in the calm/relaxed/self-accepting scale and emotion regulation (all *p*s < 0.01). No sig. findings on the RRS factors (*p* > 0.05).
		C: Juniors 16.41 (0.85)			DERS	**E**	0.13	0.20	0.18	
		100% female			Reflective pondering (RRS)	**E**	0.18	0.01	0.08	
					Moody pondering (RRS)	**E**	0.09	0.19	0.22	
					SICBC	**E**	0.10	0.24	0.13	
12. Corbett, 2011	107	8–11	Elementary school located at university campus, (Florida, USA)	M-group (*n* = 63) vs. C (*n* = 44), cortisol measures: M-group (*n* = 12) vs. C (*n* = 13)	State Anxiety (STAIC)	**E**	0.70	^#^	0^#^	ANCOVAs with pretest scores as covariates: No sig. differences between M-group and C in test anxiety, cortisol release, positive, and negative affect after the Mindfulness training (all *p*s > 0.05).
		9.94 (0.76)			TAS-C	**E**	0.52	0.11	−0.63	ANOVA on STAIC difference scores showed no sig. difference between groups in level of reported state anxiety (*p* > 0.05). ANOVA on pop quiz scores demonstrated no sig. difference between groups (*p* > 0.05).
		4th + 5th grade			PANAS-C	**R**	0.37	0.07	−0.43	
		47% female			CCTT	**C**	−0.50	0.84	1.18	
					Pop quiz	**–**	−0.37	1.06	−0.44	
					Salivary cortisol	–	−0.74	0.02	0.14	
13. Frenkel et al., in press	47	13–15	Private secondary school (Germany)	M-group (*n* = 24) vs. waitlist C (*n* = 23) Classes had been assigned randomly to conditions, follow up after 6 weeks.	Test d2	**C**	0.04	1.48	−0.06	MANOVAs: marginally sig improvement in combined parents ratings (*p* = 0.071) and measures of cognitive performance (*p* = 0.067).
		14.59 (0.54)			Unnoticed Mind Wandering	**C**	0.20	0.13	0.15	ANOVAs: M-group demonstrated sig. decrease in mind wandering noticed by others (*p* < 0. 05) which sustained in f –up (*p* < 0.10). Subjects in M-group were more likely not to notice their Mind Wandering (self-noticed Mind Wandering *p* < 0.10).
		9th grade			Mind Wandering noticed by others	**C**	−0.86	0.84	1.26	
		46% female			Self-noticed Mind Wandering	**C**	0.11	0.35	0.38	
					PSQ	**S**	0.42	0.22	−0.12	
					Kiddo-KINDL-R	**R**	−0.23	0.06	−0.11	
					PANAS	**R**	0.03	0.11	−0.18	
					KINDL (parents)	**T**	0.38	0.35	−0.35	
14. Hennelly, 2011	99	11–17	3 typical, mixed-gender state secondary schools (UK)	M-group (*n* = 53) vs. C (*n* = 46), follow-up after 6 months	WEMWBS	**R**	−0.11	0.19	0.41	ANOVAs and pairwise comparisons by age, gender and group: Sig. effects on well-being due to decreasing scores of C, while participants scores remained steady (*p* < 0.05). In Ego-Resilience only the oldest students of M-group (12 Grade) reported sig. improvement (*p* < 0.05). Female participants ego-resilience increased compared to female controls whereas male participants ego-resilience reduced. At post-test, female participants scored sig. higher on ERS than male participants (*p* < 0.01). Compared to post-test, M-group showed a further increase of well-being and a slight decrease of ego-resilience at follow up.
		7th-12th grade			ERS	**R**	0.53^#^	0.04^#^	0.08^#^	
		50% female								
15. Huppert and Johnson, 2010	134	14–15	2 independent, fee-paying boys schools, 5% ethnic minorities (UK)	M-group (*n* = 78) vs. C (*n* = 56)	ERS	**R**	−0.08	0	0	Multiple regressions: no sig. overall differences between M-group and C for resilience (*p* < 0.05). Condition was found to contribute marginally significantly to change in well-being (*p* < 0.01). Sig. improvement of well-being related to the degree of individual practice (*p* < 0.05).
		100% male			WEMWBS	**R**	−0.09	0.26	0.34	
16. Metz et al., 2013	216	16,45 (0.95)	2 high schools in a suburban district (USA)	M-school (*n* = 129) vs. C—school (*n* = 87)	DERS	**E**	−0.11	0.42	0.26	MANOVA on mean gain scores: Sig. difference between groups (*p* = 0.003) and approximately 12% of multivariate variance of the dependent variable is associated/can be explained by with the group factor.
		10th-12th grade			Psychosomatic complaints	**E**	0.03	0.37	0.20	ANOVAs: compared to C, M-group demonstrated improvement in emotion regulation (*p* = 0.021), self-regulation efficacy (*p* = 0.001) and a lager reduction in psychosomatic complaints (*p* = 0.043). Sig. effect for several subscales of DERS and psychosomatic items (all *p*s < 0.05). M-group reported 10% decrease in amount of stress, whereas C stated no change (*p* = 0.005).
		36% female			ASRES	**R**	−0.16	0.56	0.48	
					Stress level Item	**S**	0.19	0.43	0.40	
17. Kohls and Sauer, unpublished raw data	87	9th–12th	Public secondary school	M-group (*n* = 29–31) vs. C (reading training: *n* = 24–26; passive: *n* = 22–30)	Attention test	**C**	−0.34	0.34	0.27	Analysis of Effect sizes: M-Group demonstrated improvement in Attention compared to C. Well-being scores in M-group remained stable, whereas scores in C were decreasing. No difference between groups in vulnerability to stress and physical symptoms. In psychological symptoms, M-group proved the smallest increase. Compared to C, M-group showed strongest improvement in emotion regulation in response to stress.
		5th grade	(Germany)		KINDL	**R**	−0.19	−0.02	0.47	
					Vulnerability (SSKJ)	**S**	−0.36	0.07	−0.03	
					Stress symptoms (SSKJ)	**S**	−0.32	−0.33	0.02	
					Emotion-Regulation Items (SSKJ)	**S**	0.08	0.12	0.25	
18. Schonert-Reichl and Lawlor, 2010	246	9–13	12 public elementary schools,	M-group (*n* = 139) vs. waitlist C (*n* = 107)	Optimism (RI)	**R**	^#^	0.02^#^	0.27^#^	ANCOVAs on change scores: M-group showed increase in optimism (*p* < 0. 05) and positive affect (*p* < 0.10), but no decrease in negative affect. No main effect for Group on the two self-concept subscales, but sig. interaction effect for Group and Age for general self-concept: Participants in grade 4 and 5 reported sig. improvement in general self-concept, whereas controls in this age showed sig. decreases. In contrast, M-group in grade 6 and 7 demonstrated sig. decrease in self-concept and students in control condition increased.
		11.43 (1.07)	57% identified English as their first language, diverse range of socioeconomic status (Canada)	Teachers, instructing M in their classes had been assigned randomly	PANAS	**R**	^#^	0.02^#^	0.10^#^	ANCOVA on post-test scores: teacher ratings yielded an sig. intervention effect on total score in all subscales (all *p*s < 0.001).
		4th-7th grade			School self-concept (*SD*)	**R**	0^#^	0^#^	0^#^	
		48% female			General self-concept (*SD*)	**R**	0^#^	0^#^	0^#^	
					TRSC (teacher)	**T**	^#^	0.73^#^	0.73^#^^°^	
**TWO ARMED COHORT STUDY**										
19. Lau and Hue, 2011	48	14–16	2 Public schools for students with lower performance (Hong Kong)	M-group (*n* = 24) vs. C (*n* = 24)	SPWB	**R**	0.25	0.44	0.52	MANOVAs, ANOVAs and *post-hoc* tests: No sig. effect on well-being total score (*p* = 0.22), although M-group had significantly higher levels at personal growth dimension in post-test compared to C (*p* = 0.04). Sig. Time and Group interaction for combining depressive symptoms and perceived stress (*p* = 0.01). C's level of depression increased at post-test (*p* = 0.01), whereas in M-group there was no increase (*p* = 0.13).
					DASS	**E**	−0.49	0.26	0.84	
					PSS	**S**	−0.35	0.47	0.88	
**NON-CONTROLLED TRIALS**										
20. Anand and Sharma, in press	33	14.23	Public high school, middle socio-economic status, urban background (Bangalore, India)	Pre-post, follow-up after 3 months	SSS	**S**	—	1.64	—	ANOVAs: participants reported sig. reduction in perceived stress and sig. improvement in well-being from pre-test to post-test and from post-test to follow-up. Detailed analysis revealed sig changes in 5 of 7 subscales of SSS and in all of PWI-SC (no *p*s reported).
		46% female			PWI-SC	**R**		1.51		
21. Beauchemin et al., 2008	34	13–18	Private residential high school specialized in serving students with learning disorder (Vermont, USA)	Pre-post	SSRS (student)	**R**	—	0.53	—–	*T*-tests: Students reported sig. reduction in state and trait anxiety, and sig. increase in social skills (all *p*s < 0.05). Sig. improvements emerged for teacher ratings on all 3 subscales (social skills, problem behavior, and academic performance; all *p*s < 0.05).
		16.16			SSRS (teacher)	**T**		0.74		
		29% female			STAI	**E**		0.66		
22. Biegel and Brown, 2010	79	6–8	Elementary school (California, USA)	Pre-post, follow-up after 3 months	BEEDS	**R**	—	0^#^	—–	ANOVAs and *post-hoc* tests: Sig. improvement in one aspect of attention (executive control; *p* < 0.01) form pre-test to post-test. Score stabilized from post-test to follow-up (*p* = 0.86). Sig. improvement in teacher rating of social skills from pre-test to post-test (*p* < 0.05), which stabilized at follow-up (*p* = 0.75).
		2nd + 3rd grade			Sense of Relatedness scale	**R**		0^#^		No other results reported.
					Altering (ANT-C)	**C**		0^#^		
					Orienting (ANT-C)	**C**		0^#^		
					Executive Control (ANT-C)	**C**		0.41^#^		
					SSRS (teacher)	**T**		0.16^#^		
23. Joyce et al., 2010	141	10–13	2 primary schools in Melbourne's outer suburbs (Australia)	Pre-post, sample size varied between Questionnaires	Total Difficulties (SDQ)	**E**	—	0.26	**—**	*T*-tests: Participants showed sig. reductions in total difficulties score of SDQ (*p* < 0.00). On the prosocial scale, only students with initially low scores demonstrated sig. enhancement (*p* < 0.05). Further, students proved sig. reductions in depression levels due to large changes in high-scoring individuals (*p* < 0.01).
		11.4		CDI: 120;	Prosocial behavior (SDQ)	**R**		0.15		
		5th + 6th grade		SDQ Diff.: 129; SDQ Prosoc.: 141	CDI	**E**		0.27		
		44% female								
24. Wisner, 2008	28	15–19	Public alternative high school in a small city.	Pre-post	BERS-2/Teacher rating scale	**T**	—	0.83	—	*T*-tests: According to teacher ratings, students showed sig. improvement on behavioral and emotional functioning (*p* < 0.001). A sig. increase was also revealed in each subscale (all *p*s < 0.05). ANOVAs: No interaction effects on gender, grade level, and age.
		17.86	At risk of dropping out of school. (USA)							
		10th-12th grade								
		38% female								

# Data essential for exact calculation of effect sizes were not provided. If possible we appraised effects based on information given, as graphs for example.

° Teachers rated improvement form pre- to post-test after the training in M-group and Control. Between group differences were used to estimate within effect sizes as well as effect sizes of change scores.

SD, Standard deviation; M-group, Mindfulness-group; C, Control; RCT, Randomized controlled trial; ANOVA, Analysis of variance; ANCOVA, Analysis of covariance; MANOVA, Multivariate Analysis of Variance; MANCOVA, Multivariate analysis of covariance Domains: C, Cognitive Performance; E, Emotional Problems, R, Factors of Resilience; S, Perceived Stress and Coping; T, Third Person Rating. Measures: ACTeRS, ADD-H Comprehensive Teacher Rating Scale; ANT-C, Attention Network Test for Children; ASRES, Affective Self-Regulatory Efficacy Scale; AURE, Self-Concept and Self-Actualization Questionnaire; BEEDS, Behavioral and Emotional Engagement vs. Disaffection scale; BERS-2, Behavioral and Emotional Rating Scale; BRIC, Behavior Rating Index for Children; BRIEF, Behavior Rating Inventory of Executive Function; CCTT, Children's Color Trail Test; CDI, Children's Depression Inventory; DASS, Depression Anxiety Stress Scale; DERS, Difficulties in Emotion Regulation Scale; EP, Emotion Profile Inventory; ERS, Ego-Resiliency Scale; FBS, Feel Bad Scale; KINDL, QoL Questionnaire for Children and Adolescents; MASC, Multidimensional Anxiety Scale for Children; PANAS-C; Positive and Negative Affect Scale for Children; PIML, People in My Life; PSS, Perceived Stress Scale; PWI-SC; Personal Wellbeing Index—School Children; RRS, Ruminative Response Scale; RSQ, Responses to Stress Questionnaire; SCSI, Schoolagers' Coping Strategies Inventory; SD, Self-Description Questionnaire; SDQ, Strengths and Difficulties Questionnaire (Diff., difficulties subscales; Prosoc., prosocial behavior subscale); SICBC, Somatization Index of the Child Behavior Checklist; SMFQ-C, Short Mood and feelings Questionnaire—Child Version; SPPC, Self-Perception Profile for Children (Global Self-Worth Subscale); SPWB, Scales of Psychological Well-Being; SSKJ, Stress and Coping Questionnaire for Children and Adolescents; SSRS, Social Skills Rating System; SSS, School Situation Survey; STAIC, State-Trait Anxiety Inventory for Children; TASC, Test Anxiety Scale for Children; TEA-Ch, Test of Everyday Attention for Children; TIPI, Ten Item Personality Inventory; TTCT, Torrance Test of Creative Thinking; WEMWBS, Warwick-Edinburgh Mental Well-being Scale.

Two kinds of overall ESs were estimated. First, a within-group effect size was derived, based on the average of pre-post changes of intervention group in every study. Second, a controlled between-group effect size was calculated for all controlled trials. It was based on average change score differences between intervention group and control. A change score comparison was chosen instead of a simple post-test comparison, because baseline equivalence could not be assumed for all studies, and this might bias the estimation of intervention effects.

Standard errors of within group and controlled effect sizes were calculated according to the following formulas:

(2) $$\begin{array}{l} SE_{within\;group}=\sqrt{\frac{1}{n}+\frac{g^{2}}{2(n-1)}}\text{ and}\\ \;SE_{controlled}=\sqrt{\frac{n_{1}+n_{2}}{n_{1}n_{2}}+\frac{g^{2}}{2(n_{1}+n_{2})}} \end{array}$$

Initially, we grouped ES into four domains which had been shown to be affected by mindfulness practice in adults according to measurement method and construct: perceived stress and coping (S), factors of resilience (R), and emotional problems (E) were measured via self-report scales. A domain of cognitive performance (C) was measured by performance tests. Subsequently, given that a lot of studies used questionnaires for parents and teachers addressing various domains, we created a fifth domain containing third person ratings (T) exclusively. Independence of results was ensured for all analysis. Where a study contributed several ES to the same domain, ES were averaged.

Reliability of measures could not be used to adjust effect-sizes, as authors did not consistently report reliability and the measures that were reported were not compatible with each other.

The inverse variance random-effects model (DerSimonian and Laird, 1986) was chosen to carry out quantitative synthesis. This model incorporates an assumption that the population parameters vary from study to study. As a consequence, variation in effect sizes are not only caused by sampling error, but also occur due to differences between hyperparameter and population parameter values. Thus, results can be generalized beyond the included studies. The between-study variance tau-squared (τ^2^) is the estimated standard deviation of underlying effects across studies.

Heterogeneity between studies was assessed via the *Q* and the *I*^2^ statistic. The *Q*-test determines the probability of sampling errors being the only cause for variance. Under the hypothesis of homogeneity among effect sizes, the *Q* statistic follows the chi-square distribution. As a result, significant *Q*-values can be considered as evidence for heterogeneity because variance is also due to differences between effect sizes. The *I*^2^ index describes the percentage of the variability in effect estimates that is caused by heterogeneity. *I*^2^ of around 25, 50, and 75% would be interpreted as low, medium, and high heterogeneity. To identify publication bias a funnel plot was used. A funnel plot is a scattergram where the ES is plotted at the horizontal axis and the study size is plotted on the vertical axis. With no availability bias, one should see a funnel turned upside down. In case of bias, when smaller studies without significant effects were not available, the scattergram should deviate noticeably from the symmetrical funnel shape. Additionally we used the fail-safe *N* as a rough measure of the robustness of our analysis against availability bias. The fail-safe number (*k*_*fs*_) estimates the number of unavailable null result studies that would be required to render the overall *p* level of the meta-analysis insignificant. If the fail-safe number is large (larger than 5k + 10), essential influence of bias on mean effects of meta-analysis are unlikely (Rosenthal, 1991).

### Feasibility

When a new intervention has just been implemented, information on feasibility of the process is a rich source for improvement, refinement, and adaptation of the intervention at later stages. The term *feasibility* here is understood as assessing the applicability of the different programs, their strengths, and weaknesses. For this analysis of the data we assumed two different areas of focus (Bowen et al., 2010): (1). *Acceptability:* to what extent the program is judged as suitable, satisfying, or attractive to program deliverers (teachers) and recipients (students). (2). *Implementation:* to what extent the program is successfully delivered to intended participants in the context of daily school-routine.

## Results

### Trial flow

**Figure 1:**
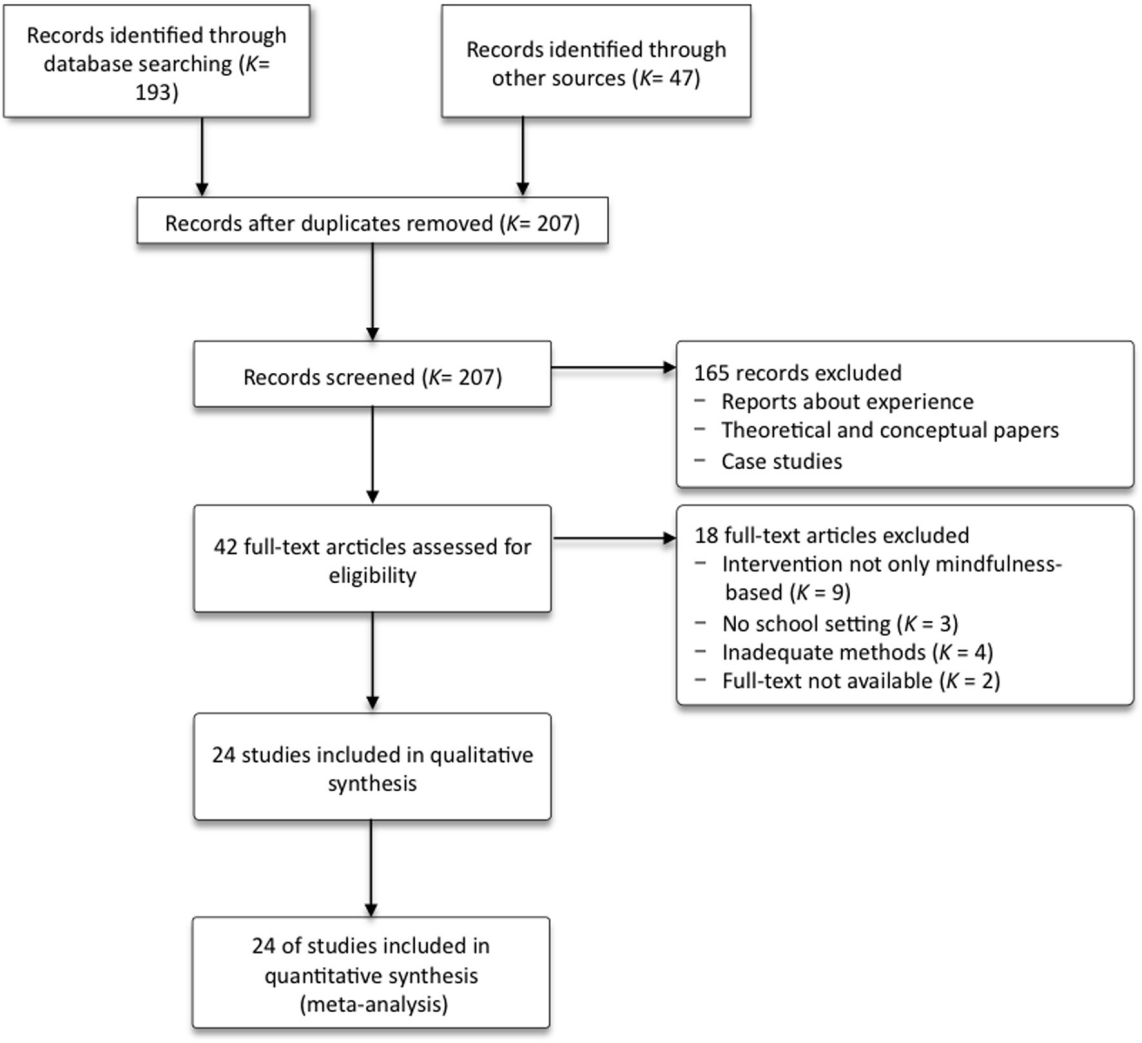
**Flow of information from identification to inclusion of studies**.

In Figure 1, the study selection process is visualized in a PRISMA flow diagram (Moher et al., 2009). The initial search provided 207 possibly relevant records after duplicates were removed. One hundred and sixty-five records were excluded after screening, mostly because they were reports or conceptual papers rather than experimental or scientific studies. Further screening of 42 full manuscripts against inclusion criteria identified 24 studies. The most prevalent reasons for exclusion at this stage were that the intervention could not clearly be defined as solely mindfulness-based (*K* = 9), but was combined with relaxation techniques such as Progressive Muscle Relaxation, visualization, or bio-feedback. Further, three studies were excluded because the intervention was implemented in a setting other than regular school life, such as a summer camp for example. Finally, four studies did not meet methodical criteria as they used an ideographic approach (*K* = 2) or were case studies (*K* = 2). Authors of two unpublished studies which had been identified as potentially relevant in the second screening did not provide the full-text article or data (*K* = 1), or could not be reached (*K* = 1). Qualitative and quantitative syntheses are based on all 24 studies.

### General study characteristics

Study characteristics are outlined in Table 1. Of the 24 studies that had been located, 13 were published in a peer-reviewed journal, and three were in press. Unpublished studies comprised manuscripts published on the internet (*K* = 2), unpublished data (*K* = 1), or Master's (*K* = 2) and PhD dissertation theses (*K* = 3). The earliest study was published in 2005. Fourteen studies were carried out in North America, seven in Europe, one in Australia, and two in Asia. In total, 1348 students were instructed in Mindfulness, and 876 served as the comparison group, ranging from grade 1–12, reflecting age 6 to 19. Sample sizes of studies varied between 12 and 216. Studies differed greatly in how they described the setting, intervention, and sample.

In eight studies, mindfulness training was implemented at elementary school level (grade 1–5), in two studies at middle school level (grade 6–8), and in 14 studies at high school level (grade 9–12). In one study, mindfulness was introduced to students from grade 7–12. In most studies, description of school, neighborhood, or participants was very limited. There was a wide variety of school types, including mostly public schools (urban and suburban), a private residential school, a catholic school for girls, a fee-paying boys' school, a rural high school, and a public alternative high school. Where sample characteristics were mentioned, samples were mostly of low socio-economic status and students were described as low performing or “at risk.” However, it is very probable that other samples might be from higher socio-economic backgrounds, which would result in a diverse range of sample characteristics (see Table 1).

### Interventions

The programs of this database have been reviewed and rated into different domains according to underlying theory, objectives, components, and intensity. If an intervention is to be evaluated in terms of effectiveness, it is necessary that details of the program, such as the theoretical base, well defined goals, explicit guidelines, training, and quality control, are described (Weare and Nind, 2011) and steps of implementation are carefully documented (Durlak and DuPre, 2008). Not all of the studies offered sufficient information on program details or implementation, and some additional work was necessary to gather sufficient information. This part of the analysis will be reported in another article (Herrnleben-Kurz et al., in preparation). Here we summarize basic details about interventions and programs.

As can be seen in Table 2, the *theoretical framework* of the programs refers to the concept of mindfulness. In most cases theory is linked to previously existing mindfulness programs, such as MBSR, MBCT, DBT, and ACT. Some interventions also make reference to theories and findings from positive psychology, or combine MBI with a special group of school-based intervention programs, such as social and emotional learning (SEL).

**Table 2:** **General features of MBI's applied**.

**General features**	***K***	***%***
**THEORETICAL FRAMEWORK**		
Mindfulness	24	100
Positive psychology (including SEL)	9	38
Executive function	6	25
**USE OF PROGRAM MANUAL**		
Existing since > 5 years (≤2007)	2	8
Existing since < 5 years	13	54
*Ad-hoc* program	9	38
**INTERVENTION FEATURES**		
Class by teacher	7	29
Class by non-school trainer	15	63
Class by teacher and non-school trainer	2	8
**INTERVENTION COMPONENTS**		
Breath awareness	24	100
Working with thoughts and emotions	21	88
Psycho-education	20	83
Awareness of senses and practices of daily life	20	83
Group discussion	18	75
Body-scan	14	58
Home practice	12	50
Kindness practices	11	46
Body-practices like yoga	6	25
Mindful movement (≠ other body-practices)	5	21
Additional material	10	42

Manualized programs, such as *MindfulSchools* or *Learning to BREATHE*, were identified in two thirds of the studies. These programs were generally available but only two had an enduring presence of more than five years, and many did not contain sufficient guidance material for implementation. Others were reported to be manualized, but the material was not made available (see Table 2). The programs themselves often define similar *objectives*. These are mostly related to the assessment methods and mirrored in the domains which have been identified (see outcome methods below).

Most programs contain more than one *component* to facilitate mindfulness, with observation of breath as the traditional essential exercise, as well as psycho-education and group discussions (see Table 2).

Predominantly, MBIs were conducted by professional trainers, most of whom were involved as study authors. Few interventions had been instructed by the class teachers, and not all had personal experiences with mindfulness practices. Some had briefly been introduced to the topic, while others had undergone a MBSR course before implementation.

The periods and *intensity* (frequency and length) of training varied from 4 weeks to 24 weeks with a median of 8 weeks, with 45 min once a week in most programs. Some programs split this over several sessions per week. In total, interventions varied from 160 to 3700 min of practice, with a median of 420 min.

### Study quality assessment

As can be seen in Table 1, 19 of the 24 studies used a controlled design and five used a pre-post design. Randomized designs were realized in studies where mindfulness training was offered as an alternative or extracurricular activity at school (*K* = 10). Students who signed up for the mindfulness training were randomly allocated to either a mindfulness or control group. In one study, a group of students with matched backgrounds was invited to function as control. In quasi-experimental designs, mindfulness was taught in a classroom setting and another class, mostly the parallel class, served as control (*K* = 8). In another study (Study 17, Table 1) a reading training of the same intensity as the MBI took place. Selection and allocation of classes to interventions was mainly decided upon by the heads and classroom teachers. In four studies, classes or schools were randomly assigned to conditions. Follow up measures were collected in five studies.

For every effect size we performed a *post-hoc* power analysis using the software program G*Power (Faul et al., 2009). Given an alpha of 0.05 (one-sided), and a power of 80%, a sample size of *n* = 41 was determined for pre-post ES to detect an effect of *d* = 0.40. Twelve studies met this criterion. The same procedure for controlled ES revealed a sample size of *n* = 78 per group, which was achieved in three controlled studies.

Fifteen studies reported data on attrition in the intervention group, in which rates varied between 0% (23) and around 40% (1, 19), either due to invalid or incomplete data (7, 10, 11, 12, 13, 17, 23), or because students did not fulfill a defined amount of attendance or home practice (1, 5, 6, 8, 19). Eight studies specified reasons for withdrawal, mostly naming scheduling conflicts, school transfers, or school absence. Two studies reported drop-outs due to parental refusal (12, 16) and in one case five students decided to leave the training after the first session (19).

### Outcome measures

A variety of measures were applied to investigate the effects of mindfulness training. We grouped the outcomes into the domains as follows:

#### Cognitive performance (C)

Nine measures in total were classified in the domain of cognitive performance. In most cases, cognitive performance was quantified by attention tests (Studies 8, 12, 13, 17, 22, Table 1). A creativity test (3) was used in one study, and in another (13) the mind wandering paradigm was applied. Two studies (4, 6) used grades as dependent variables.

#### Emotional problems (E)

In the domain of emotional problems self-report questionnaires focusing on maladaptive emotion, cognition, and behavior are summarized, also including clinical symptoms, such as anxiety and depression (4, 7, 9, 12, 19, 21, 23), test anxiety (8, 12), somatic reactions (11, 16), ruminative thinking style (11) emotion regulation difficulties (6, 9, 11, 16), and various difficulties (23).

#### Stress and coping (S)

Nine Studies investigated changes of perceived stress and coping behavior via self-report questionnaires (7, 9, 10, 13, 16, 17, 19, 20). In one study (12) cortisol measures in combination with a stress test (math quiz) were carried out. These outcomes were examined separately.

#### Resilience (R)

Seventeen studies collected self-report data on constructs we categorized as factors of resilience: well-being (13, 14, 15, 17, 19, 20), positive and constructive emotions or affect (7, 11, 12, 13, 16, 18, 22), resiliency (14, 15), social skills and positive relationships (7, 21, 22, 23), self-concept and self-esteem (4, 5, 10, 18).

#### Third person ratings (T)

In the domain of third person ratings, parent and teacher questionnaires were grouped, dealing with aspects such as aggressive or oppositional behavior, social skills, emotional competence, well-being, attention, and self-regulation (1, 2, 6, 8, 13, 18, 21, 22, 24).

**Figure 2:**
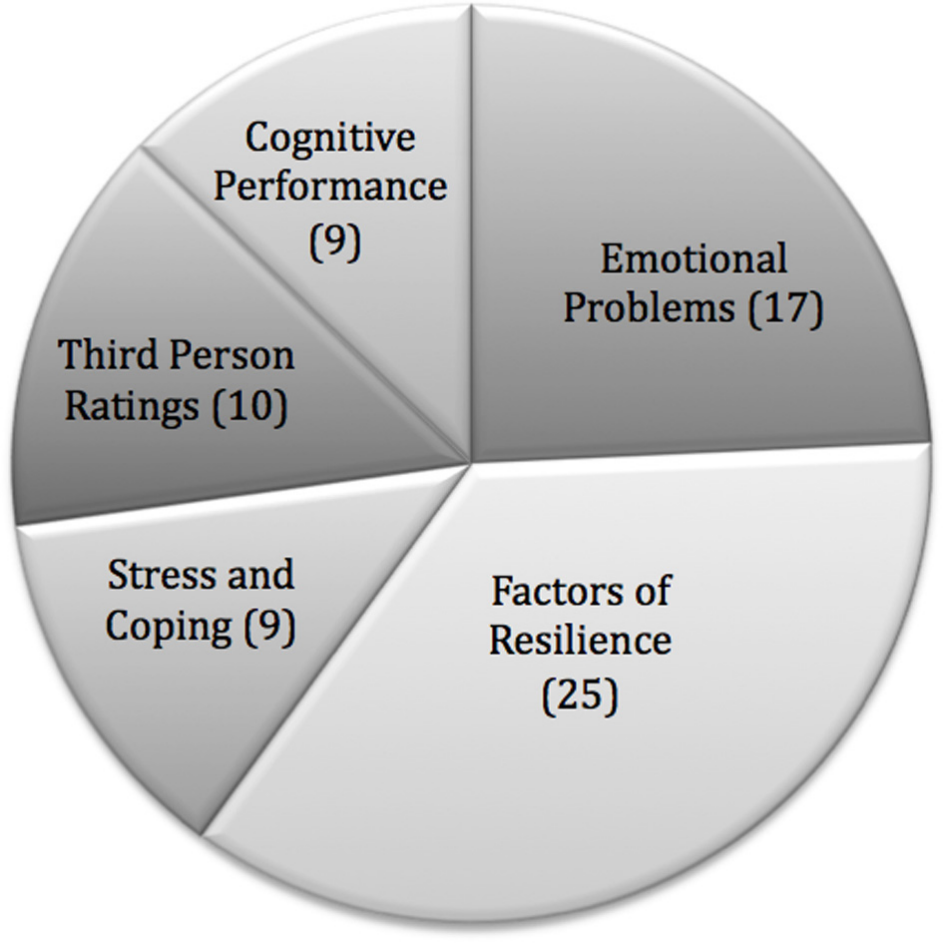
**Numerical proportions of measures applied in studies**.

Another study measured school attendance (6). Since this measure does not fit any of the domains, it was not included in the domain-specific analyses. The numerical proportions of measures applied in studies are portrayed in Figure 2.

### Feasibility

Only some of the studies offered information about how the integration of the program into school-routine was working. In some studies, one or more aspects of feasibility were assessed systematically via questionnaires, focus groups, or interviews. Some reported a systematic assessment, but did not provide a report or an analysis of respective data. Others reported only anecdotal evidence.

### Acceptability

One third of studies provide information about acceptability. There seems to be an overall high acceptability in those studies referring to students and teachers, but, again, methods were partly heterogeneous and unsystematic.

Results of interviews and focus groups (teachers and students) indicate a uniformly positive experience of the intervention (Beauchemin et al., 2008; Mendelson et al., 2010; Lau and Hue, 2011). Eighty-nine per cent of the students would recommend the training to others (Broderick and Metz, 2009; Metz et al., 2013). In Anand and Sharma's study (in press) 81% of the students rated the program sessions as extremely useful, and 83% as satisfying.

Three quarters of the students said that they would like to continue, and thought that it could have lasted longer (Beauchemin et al., 2008; Huppert and Johnson, 2010), or that it was the right length (Anand and Sharma, in press). Only 5% thought that the intervention was too long (Huppert and Johnson, 2010). Potek (2012) cited a noteworthy statement: *“We just started getting it. I think we should have more time to practice.”*

Some of the programs also contain an individual *home practice*: Huppert and Johnson (2010) found that one third practiced at least three times a week and two thirds once a week or less. In Broderick and Metz's study (2009), two thirds of the participants practiced mindfulness techniques outside the classroom. By analyzing the protocols, Frenkel et al. (in press) found that no one practiced the full amount of weekly exercises and two thirds failed to do their homework at least once.

### Implementation

Joyce et al. (2010) mentioned specific factors which facilitated successful implementation: teaching along with colleagues, administrative and parental support, or children's enthusiasm. What hindered was a lack of time and students who failed to engage with the program. In the study of Beauchemin et al. (2008), teachers suggested that the intervention was feasible when conducted in a classroom with voluntary participation. Desmond and Hanich (2010) mentioned problems regarding scheduling, completion of administration, beginning of holidays, and difficulties with participants arriving too late. Some studies provided information about feasibility of different program-elements, and very few reported *implementation integrity* which had been assessed via protocols, detailed scripts, feedback formulas, or fidelity logs. Because these data were rare we did not include them in the analysis of outcomes.

### Quantitative synthesis

#### Within-group effect size

**Figure 3:**
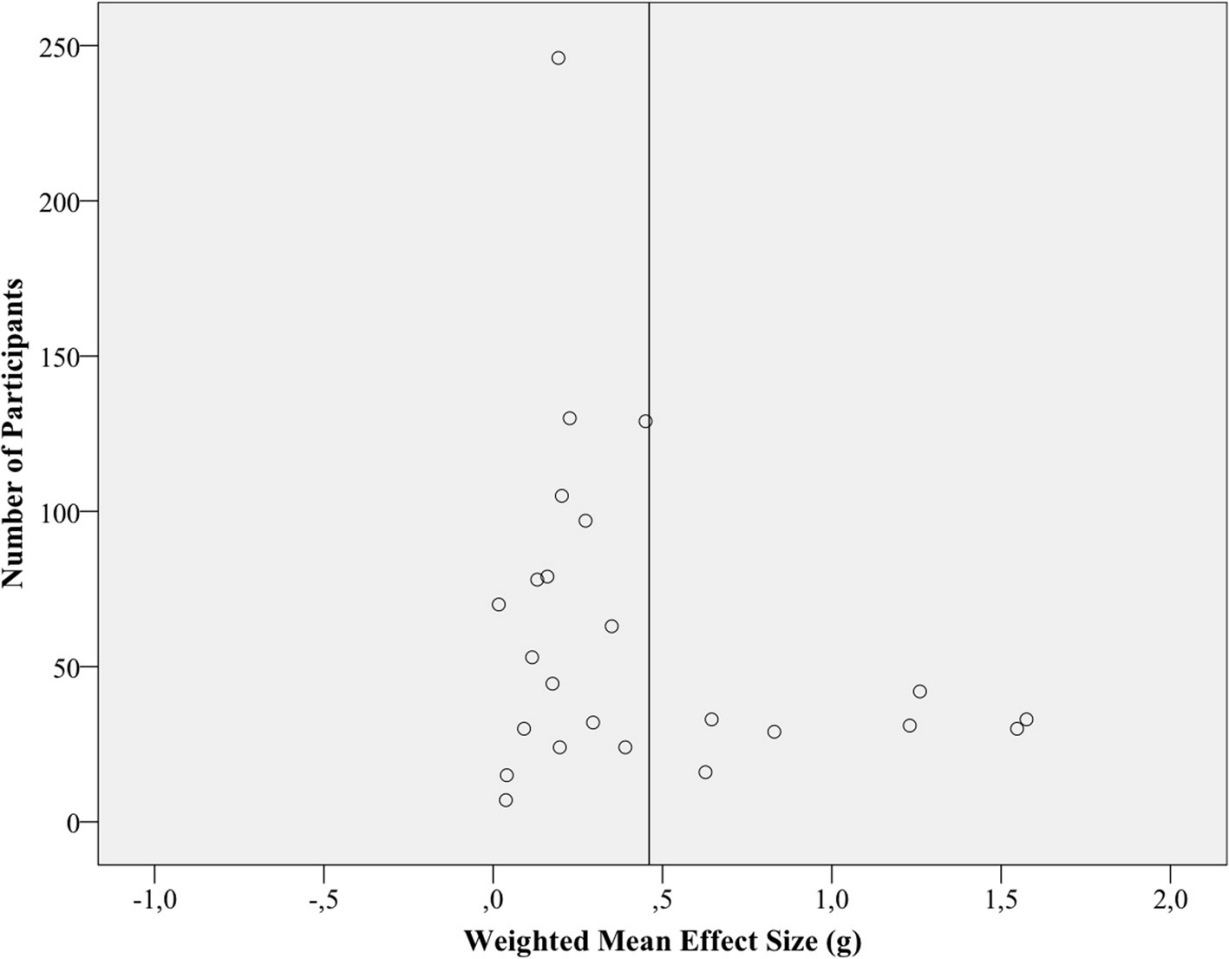
**Funnel plot of within-group effect sizes (*K* = 24)**. The vertical bar represents the weighted (by sample sizes) mean effects size.

The results of the quantitative synthesis are reported in Table 3. Weighted mean effect sizes for within-group effect sizes was *g* = 0.41 (95% CI 0.28–0.54), which can be considered as a small to medium effect. The *Q* statistic indicates heterogeneity, and the *I2* index shows that a large amount of variance is caused by it. The fail-safe number exceeded the criterion. Figure 3 shows a funnel plot of the respective 24 effect sizes where the vertical bar marks the weighted mean effect size. Asymmetry can be seen: Studies with small sample sizes and small or even negative effects are lacking. Only a few studies, with rather small sample sizes, are located above the estimated mean effect size. Sensitivity analyses, excluding the five studies with partly estimated ES (#) from synthesis, lead to slightly higher ES (*g* = 0.49; 95% CI 0.31, 0.67) and more between study variance (τ^2^ = 0.12). Synthesis only of studies with a minimum sample size of 41 (*K* = 12) revealed an ES of.31 (95% CI 0.18, 0.44) and a tau-squared of 0.04.

**Table 3:** **Overall within-group and controlled effect sizes and respective subgroup effect sizes, including effect size statistics**.

**Type of effect size**	**Sample**		**Effect size**			**τ^2^**	**Homogeneity**			***k*_fs_^a^**	**Criterion**^b^****
	***K***	***n***	**Hedges's *g***	**95%—CI**	***p***		***Q***	***p***	***I^2^***		
**Within-group effect**	24	1348	0.41	(0.28, 0.55)	<0.00001	0.08	112.52	<0.00001	80%	1008	130
Excluding estimated ES (#)	19	917	0.49	(0.31, 0.67)	<0.00001	0.12	104.86	<0.00001	83%	912	105
Excluding studies *N* < 40	12	990	0.31	(0.18, 0.44)	<0.00001	0.04	42.77	<0.00001	74%	360	70
Subgroup Franco	3	103	1.32	(1.05, 1.59)	<0.00001	0.00	0.92	0.63	0%	393	25
Subgroup rest	21	1245	0.29	(0.19, 0.40)	<0.00001	0.03	53.68	<0. 0001	63%	588	115
**Controlled effect**	19	1897	0.40	(0.21, 0.58)	<0.0001	0.11	59.35	<0.00001	70%	722	105
Excluding estimated ES (#)	16	1445	0.45	(0.23, 0.68)	<0.0001	0.14	54.83	<0.00001	73%	704	90
Excluding studies *n* < 77	3	656	0.31	(0.15, 0.46)	0.0001	0.0	0.10	0.95	0%	90	25
Subgroup Franco	3	205	1.34	(1.04, 1.65)	<0.00001	0.00	1.83	0.40	0%	399	25
Subgroup rest	16	1692	0.23	(0.13, 0.33)	<0.00001	0.00	11.05	0.75	0%	352	90

K, number of studies; N/n, number of participants; g, weighted mean effect size; CI, confidence interval; τ*^2^*, variance component; p, level of significance; Q, Q—Statistic.

a k_fs_ is the number of unavailable studies with null results, that would be required to reduce the overall result to an insignificant level.

b If k_fs_ is exceeding the criterion (5k + 10), an essential influence of availability bias is unlikely.

#### Controlled effects sizes

**Figure 4:**
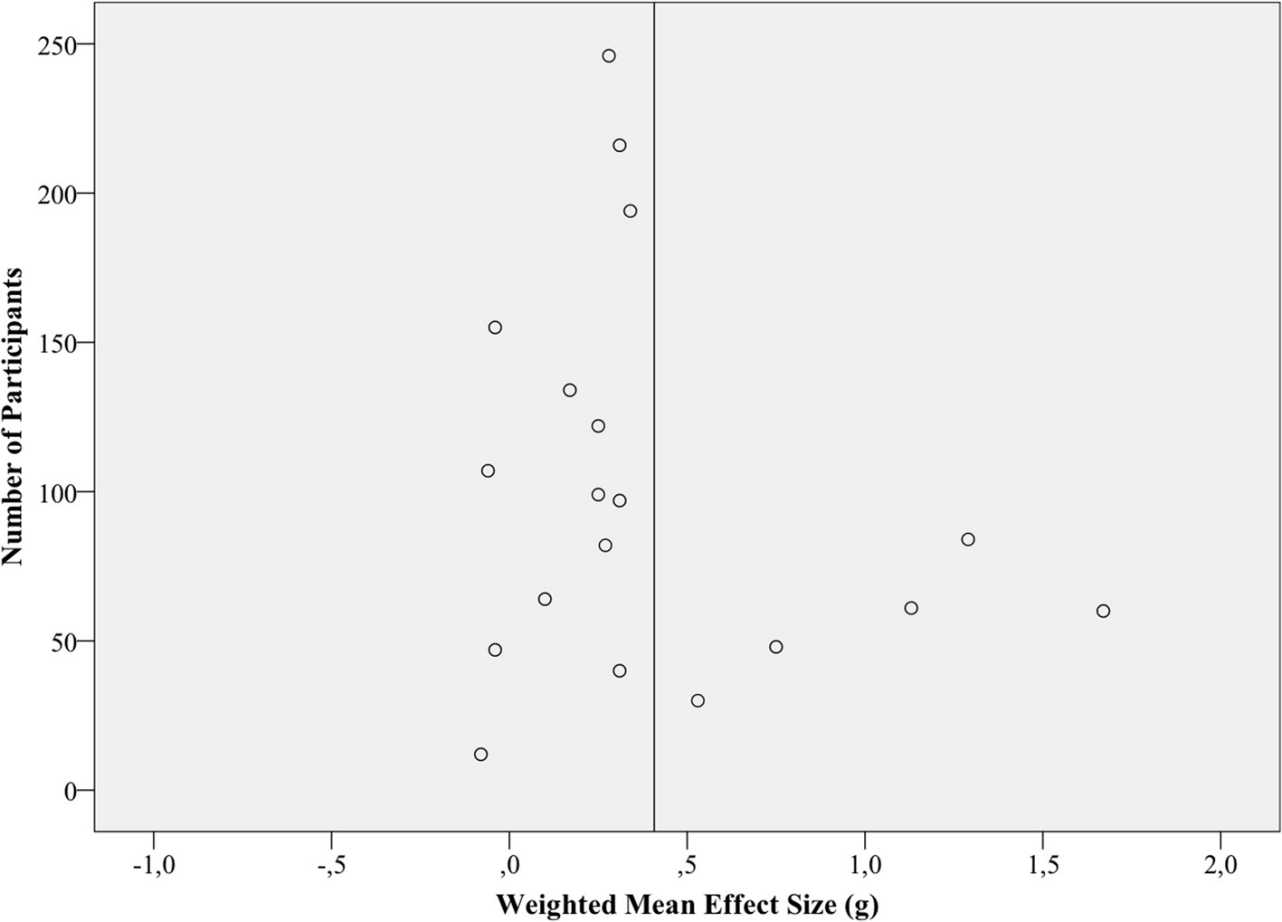
**Funnel plot of all controlled effects sizes (*K* = 19)**. The vertical bar represents the weighted (by sample sizes) mean effect sizes.

Weighted mean effect size of the 19 studies using a controlled design was *g* = 0.40 (95% CI 0.21, 0.58), a small to medium effect. Again there was evidence for heterogeneity. The fail-safe *N* criterion is exceeded. The funnel plot follows a similar pattern of asymmetry as in pre-post effect sizes, which can be seen in Figure 4. On the other hand, the fail-safe number of 722 exceeded clearly the criterion (105), indicating the robustness of results concerning availability bias. Sensitivity analyses excluding estimated ES (#) showed a similar ES (*g* = 0.44; 95% CI 0.23, 0.68) and a larger between study variance (τ^2^ = 0.14). Synthesis only including studies with an adequate ES of *n* = 78 or higher (*K* = 3) yielded a lower ES (*g* = 0.31; 95% CI 0.15, 0.46) and no between study variance (τ^2^ = 0.00).

#### Exploratory analyses

Examining ES and plots, the three studies from the Franco Justo research group were categorized as one subgroup. In three independent studies, the effects of the *Meditación Fluir* program were explored. This very sophisticated, demanding, and well-established program for graduating high-school students clearly differentiates itself from other interventions by a very high intensity. A subgroup analysis was performed for within-group effect size and controlled effect size. Separate analysis leads to a slight reduction of heterogeneity in within-group effect sizes and to complete reduction of heterogeneity in controlled effect sizes (see Table 3). In both cases CI intervals do not overlap, and the percentage of genuine subgroup differences is 98%. Differences of subgroup effects were significant for within-group effects sizes (χ^2^ = 50.21, *p* < 0.00001) and controlled effect sizes (χ^2^ = 46.47, *p* < 0.00001).

**Figure 5:**
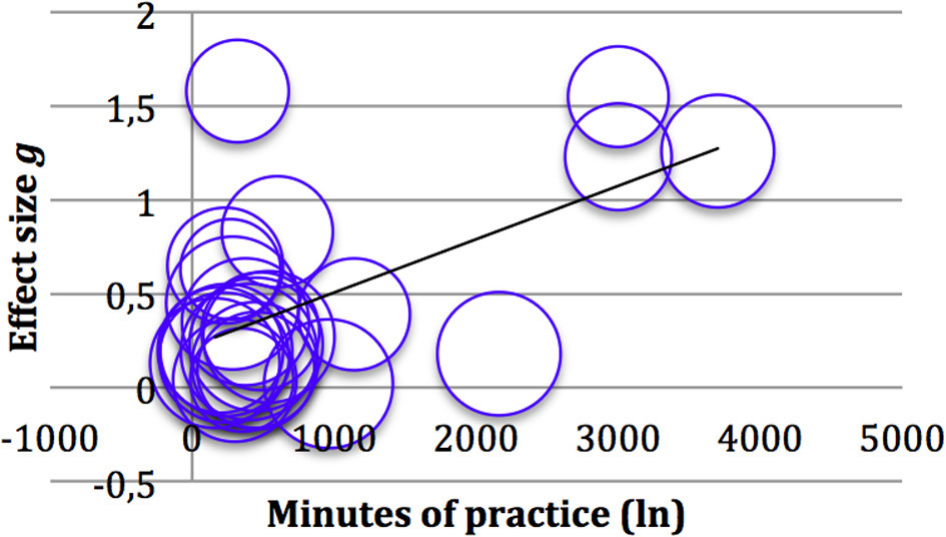
**Bubble plot of the 24 within group effects sizes against Intensity of mindfulness Training and regression line**. *R*^2^ (adjusted) = 0.21.

**Figure 6:**
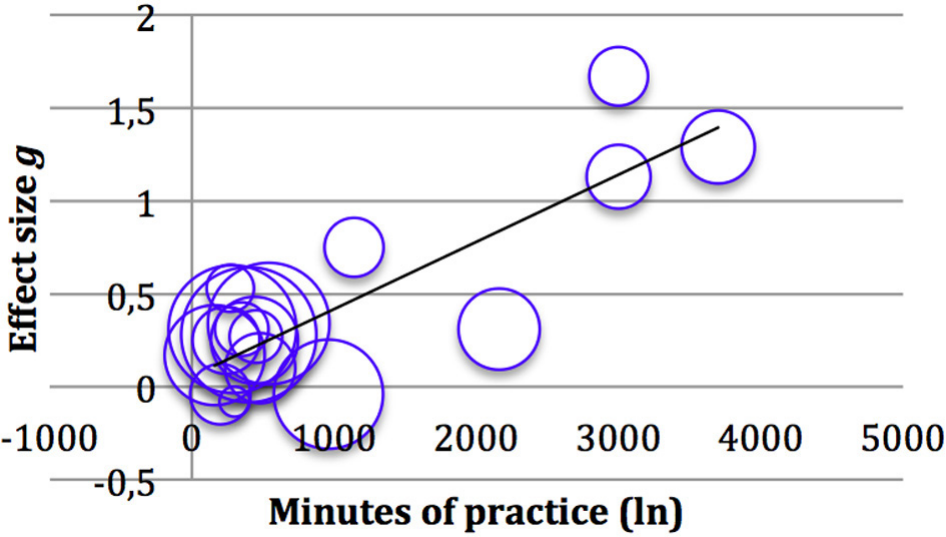
**Bubble plot of the 19 controlled effects sizes against Intensity of mindfulness training and regression line**. *R*^2^ (adjusted) = 0.52.

To investigate whether the intensity of mindfulness training explains part of the heterogeneity between ES of all studies reviewed, a random-effects meta-regression was performed. Minutes of mindfulness practice in total (including training sessions and home practice, if it was compulsory) were entered as a predictor and ES as the outcome variable. Studies were weighted by inverse variance, combining within-trial variance of treatment effect and the between study variance. As can be seen in Figures 5, 6, there is a substantial correlation between ES and minutes of mindfulness training for controlled ES, and a slightly weaker correlation for within group ES. Regression analysis shows that intensity of mindfulness practice accounts for 21% (adjusted *R*^2^ = 0.21) of heterogeneity in within-group ES and 52% (adjusted *R*^2^ = 0.52) of heterogeneity in controlled ES (see also Table 4). The three studies with the highest intensity driving the strong correlations were those from the Spanish Franco Justo research group.

**Table 4:** **Results of random-effects meta-regression on intensity of mindfulness training for within-group and controlled effect sizes**.

**Model**	***B***	**SE *B***	**Beta**	**Sig**.
**WITHIN-GROUP EFFECT SIZE**				
1. (Constant) Intensity (Min_ln)	−1.121	0.583		0.068
	0.246	0.093	0.490	0.015
**CONTROLLED EFFECT SIZE**				
1. (Constant) Intensity (Min_ln)	−1.910	0.512		0.002
	0.359	0.080	0.738	0.000

Outcomes of quantitative synthesis for each domain are presented in Table 5. Effect sizes in the domain of cognitive performance were moderate to high, whereas effect sizes of the stress and resilience domains showed small to moderate ES. The domain of emotional problems and third person ratings demonstrated small ES and CI's overlapping zero. High levels of heterogeneity could be identified in all domains except emotional problems. In the domain of emotional problems, heterogeneity was at a medium level and according to the *Q*-test, absence of heterogeneity can be assumed. The fail-safe *N* criterion was exceeded considerably in all 5 domains.

**Table 5:** **Domain specific effect sizes and statistics for within group and controlled effects sizes respectively**.

**Domain**	**Type of effect size**	**Sample**		**Effect size**		**Heterogeneity**
		***K***	***n***	**Hedges's *g***	**95%—CI**	***I***^2^****
Cognitive performane	pre-post	8	327	0.68	(0.33, 1.03)	88%
	Controlled	7	569	0.80	(0.35, 1.26)	82%
Emotional problems	pre-post	11	693	0.31	(0.19, 0.42)	44%
	Controlled	9	903	0.19	(−0.03, 0.41)	52%
Stress	pre-post	8	374	0.36	(0.05, 0.66)	85%
	Controlled	7	674	0.39	(0.07, 0.71)	78%
Factors of resilience	pre-post	17	1082	0.38	(0.20, 0.55)	86%
	Controlled	13	1497	0.36	(0.09, 0.62)	82%
Third person Ratings	pre-post	8	448	0.34	(0.08, 0.60)	84%
	Controlled	6	591	0.25	(−0.10, 0.61)	74%

K, number of studies; n, number of participants; g, weighted mean effect size; CI, confidence interval.

## Discussion

This is the first systematic review and meta-analysis to summarize data available on the effects of mindfulness-based trainings for children and youths in a school setting. Twenty-four studies were located that report a significant medium effect size of *g* = 0.40 across all controlled studies and domains. Remarkably, the ES of studies using pre-post designs only is very similar, with *g* = 0.41. The effects are strongest in the domain of cognitive performance with a large and significant ES of *g* = 0.80 for controlled studies. Effect sizes are smaller but still significant in the domains of resilience measures (*g* = 0.36) and stress measures (*g* = 0.39), and they are small and not significant for measures of emotional problems (*g* = 0.19) and third-person ratings (*g* = 0.25). In the latter two domains pre-post ES are larger, while in all other domains they are either very similar to the controlled ES or even somewhat smaller. Thus, taken from a bird's eye view, mindfulness-based training in a school context has effects that are seen mostly in the cognitive domain, but also in psychological measures of stress, coping, and resilience. Acceptance seems to be high with few reported adverse events or incidents. There were some hints that implementation was not always without difficulties. It is important to keep in mind that the analysis referring to feasibility is very limited due to methodological issues.

### Strengths

We went to great lengths to locate all relevant studies and get more detailed information from authors. Since all but two authors complied with our requests, our work is novel and complete. A third of the material included in this review is unpublished gray literature. Hence, we are confident that availability bias was comparatively small. Although the funnel plot seems to indicate such a bias, one should bear in mind that the asymmetry is mainly caused by three studies with large ES stemming from one group in Spain that have developed a very intense mindfulness training. Excluding those studies from the visual analysis of the funnel plot renders it symmetrical, thus testifying to our success at locating the most relevant studies. Also, the large fail-safe Ns show that the results are robust regarding availability bias. In most cases, more than twice the number of available studies would be needed to render the ES insignificant, a rather unrealistic assumption.

We adopted conservative quantitative estimation methods. When *SD* and Means were unavailable, ES of measures were set to zero. We corrected for baseline differences by using difference-scores as the basis of ES estimation. By using correction factors for small studies, larger studies receive more weight, and by using random-effects models the large variation is taken into account. By analyzing studies both through overall ES and domain specific ES, we tried to disentangle the maze of very diverse outcome measures employed in those studies. We took care to not inflate ES by only using one contribution per outcome measure to each study. Data were inspected carefully in terms of heterogeneity and biases and various sensitivity analyses were computed. By exploring the variation through meta-regression we were able to account for a sizeable portion of the variance through one theoretically important variable, namely the amount of practice (i.e., the intensity) implemented in the study, which accounts for 52% of the variance in the controlled studies and 21% of the variance in pre-post-design studies. Given the heterogeneity of measures, students, settings, and programs, this is a remarkable finding that suggests that one of the most important factors for the variation across studies is the amount of practice that a mindfulness based program has introduced.

### Limitations

This is simultaneously the major limitation of our findings: the heterogeneity of the studies is considerable, and hence the estimates of effect sizes, including their significance, can only have an orienting function. It is plausible that school-background, social background, and how a program is accepted within a particular school context influence its effects, yet we do not have the information necessary to explore these effects or those of other potential moderators. For instance, it is a completely different situation if pupils attend within the compulsory school framework or are willing to stay on in their free time, whether there is a classroom or workshop setting. Furthermore, it makes a difference if teachers themselves implement programs or if outside trainers come and deliver the courses. Additionally, the instructors' qualifications and their personal experience with mindfulness are surely important. A lot of this information may be decisive, yet is not available in study reports.

As is the case with any nascent field of research, the heterogeneity is also built in through the exploratory framework of most studies. In only a few cases, such as with the Franco Justo research group, were studies conducted in replication. Mostly, researchers implemented their own programs. Therefore, a variety of programs were evaluated or tested. Thus, there are no manualized consensus programs available, as is the case with MBSR or MBCT. Also, outcome measures for children are much less stable, both psychometrically and age-wise. By default, a lot of tests available for children are only partially validated, or are sometimes used in age groups where no clear validation exists. Also, some of the measures might have exhibited floor or ceiling effects, especially when clinical measures are used for groups that are within normal range. While the motivation of patients studied in clinical studies of MBSR and MBCT is comparatively easy to gauge, such a motivation is less clear for children. This source of variance was completely out of reach for us, as only one study documented motivation.

Studies are often underpowered and small. This is not a surprise, given the exploratory nature of the field. It means, however, that the findings are tentative and need to be supported by larger, more robust evaluations in groups that are representative of settings where such trainings will likely be implemented. It also means that a large proportion of the effect size is derived from studies where the study size is small and hence the variation is large. Synthesis only including studies with an appropriate sample size revealed an ES of.31 for pre-post as well as controlled ES. The decrease in ES and heterogeneity indicates that our results might be slightly biased by the “small-study effect” (Sterne et al., 2000), which leads to an overestimation of ES. As a result, an overall ES of 0.31 is a more stable estimate.

None of the studies used a strong active control. Hence the ES estimate is for an effect which has not been compared with another intervention or control. The precise role the element of mindfulness really plays is unknown, as is the extent of the effect that can be attributed to non-specific intervention factors, such as perceived group support, the specialty, and novelty of the intervention, of taking time out in school and at home, or of generic resting and relaxing. We only have one indirect indicator, and this is the strong correlation between ES and mindfulness training intensity revealed by the meta-regression.

### Comparison with other findings

This is the first analysis of its kind regarding school based MBIs, as far as we are aware. Meta-analyses have been carried out in other fields, such as the clinical effects of MBSR in adults (Grossman et al., 2004). This first analysis isolated an ES of approximately *d* = 0.5, for patients and non-patients, for physical and mental health measures alike. In a more recent meta-analysis by Eberth and Sedlmeier (2012) an ES of *r* = 0.31 was found for the effect of MBSR in non-clinical adult populations, based on a larger amount of studies (*k* = 17). Thus, effects of MBIs in non-clinical settings seem to be slightly higher in adults than in children and youth.

However, the ES we derived in this analysis are in the same range as results of other meta-analyses of school-based prevention programs. A meta-analysis of school-based social and emotional learning programs, for example, revealed an overall ES of *g* = 0.30 and an *I*2 of 91% (Durlak et al., 2011). Also, the ES of 3 domains, namely emotional problems, resilience, and third person ratings, showed similar ES compared to respective categories in larger meta-analyses of school-based prevention programs. However, effects on academic achievement were lower in other meta-analyses (Durlak et al., 2011; Sklad et al., 2012). ES of stress and coping measures were much higher (*g* = −1.51) in studies targeting stress directly than in this study (Kraag et al., 2006). Levels of statistical heterogeneity of the referred studies were about the same magnitude as in our study.

### Suggestions for further work

It is obvious that more research, especially larger and randomized studies, if possible with active controls, is needed. Also, longer follow-up measures would be appropriate, primarily to see if benefits are lasting, but also to investigate potential effects of triggering developmental steps. Besides, attrition rates, including reasons for dropout, should be reported, because relevant information regarding implementation strategies, feasibility, and contraindication might be extracted. Great consideration must be given to outcome measures. As our analysis shows, the effects of mindfulness-based interventions can be rather differentiated across domains. A lot of the scales used are not really adequate. Researchers might want to pilot their measures before using them or employ measures that have been sensitive in other studies. Further, it would make sense not to exclusively rely on self-report data and questionnaires in general, but to triangulate measures with qualitative data and behavioral measures. Using qualitative approaches, new hypotheses could be generated and other adequate methods could be developed. Manuals of the intervention studied should be made available.

To prevent unnecessary failure in implementation, studies should use a mixed-methods approach to assess outcome and acceptability, adopting methods such as written teacher reports, review sessions, individual interviews, observations of training sessions and student questionnaires and interviews. For example, Greenberg et al. (2004) have described a number of criteria such as timing, dosage and quality of sessions, student absenteeism and responsiveness, teacher experience, and commitment. It should be determined which aspects of the implementation process are most important, and what adaptations can be made without harming the integrity of the intervention. All this can only be investigated if adequate information is provided. This will allow future meta-analysts to assess sources of heterogeneity better than we were able to.

What is also clear from our study is that implementing and studying mindfulness-based interventions in schools is a promising avenue. Although not formally assessed, from our own experience and in accordance with others (Roeser et al., 2012), we suggest a good model might be to train teachers in mindfulness. They could then promote mindfulness in their pupils through teaching mindfully, and through teaching mindfulness directly in diverse settings. For if mindfulness is to be established in a school-based framework it will have to be teachers who are the agents and ambassadors of change. This might be a good resource for teachers' own resilience and prevention of burnout, in addition to being, very likely, the best way of delivering mindfulness in schools.

## Summary

Our analysis suggests that mindfulness-based interventions for children and youths are able to increase cognitive capacity of attending and learning by nearly one standard deviation and yield an overall effect size of *g* = 0.40. The effect is stronger in studies where more mindfulness training and home practice has been implemented. However, results might be slightly biased by the “small study effect.” Furthermore, the heterogeneity is large and thus further work, especially locating the origin of the heterogeneity, is needed. We suggest that larger studies using robust and well validated measures be conducted, and that active controls should be considered. The available evidence certainly justifies allocating resources to such implementations and evaluations, since MBIs carry the promise of improving learning skills and resilience.

### Conflict of interest statement

The authors declare that the research was conducted in the absence of any commercial or financial relationships that could be construed as a potential conflict of interest.
